# Intestinal microbiome-targeted therapies improve liver function in alcohol-related liver disease by restoring bifidobacteria: a systematic review and meta-analysis

**DOI:** 10.3389/fphar.2023.1274261

**Published:** 2024-01-08

**Authors:** Xin Chi, Xiu Sun, Danying Cheng, Shunai Liu, Calvin Q. Pan, Huichun Xing

**Affiliations:** ^1^ Center of Liver Diseases Division, Beijing Ditan Hospital, Capital Medical University, Beijing, China; ^2^ Beijing Key Laboratory of Emerging Infectious Diseases, Institute of Infectious Disease, Beijing Ditan Hospital, Capital Medical University, Beijing, China; ^3^ National Center for Infectious Diseases, Beijing, China; ^4^ Peking University Ditan Teaching Hospital, Beijing, China; ^5^ Division of Gastroenterology and Hepatology, NYU Langone Health, New York University School of Medicine, New York, NY, United States

**Keywords:** microbiome-targeted therapies, alcohol-related liver disease, intestinal microbiota, systematic review, meta-analysis

## Abstract

**Objective:** To systematically evaluate the efficacy of intestinal microbiome-targeted therapies (MTTs) in alcohol-related liver disease (ALD).

**Methods:** With pre-specified keywords and strategies, we searched databases including Cochrane Library, PubMed, EMBASE, CNKI, Wanfang Data, and Weipu for RCTs on intestinal MTTs in ALD patients from January 2000 to May 2021. Two researchers independently conducted literature screening, data extraction, and quality evaluation according to the eligible criteria. Outcomes of interest included the effects of intestinal MTTs on ALT, AST, GGT, TBIL, TNF-α, IL-6, intestinal *Escherichia* coli, and *Bifidobacteria* when compared to the control group. Pooled data were compiled and analyzed with Revman 5.4 software.

**Results:** Among 5 RCTs included with 456 ALD patients who received probiotics, the therapeutic pooled effects in the experimental group were the followings: ALT (MD = −7.16.95% CI: 10.71∼-3.60; *p* < 0.0001)、AST (MD = −25.11.95% CI: 30.57∼-19.47; *p* < 0.00001)、GGT (MD = −6.72.95% CI: 11.91∼-1.53; *p* = 0.01)、IL-6(SMD = −0.82.95% CI: 1.10∼-0.54; *p* < 0.00001), which were significantly better than those in the placebo or standard treatment group respectively, while the difference of TBIL (SMD = −0.06, 95%CI: 0.29–0.16; *p* = 0.59), TNF-α(SMD = −0.53.95% CI: 1.57–0.50; *p* = 0.31)in the two groups was not significant. After intestinal MTT treatment, the number of intestinal *Bifidobacteria* increased significantly (MD = 0.79.95% CI: 0.00–1.58; *p* = 0.05)in the experimental group. However, there were no significant changes in the number of *E. coli* in both groups (SMD = −0.29.95% CI: 0.92–0.34; *p* = 0.36).

**Conclusion:** Intestinal MTTs can significantly improve liver function, associated with the increase of intestinal *Bifidobacteria*, which may be beneficial to ALD.

**Systematic Review Registration:** https://www.crd.york.ac.uk/prospero/display_record.php?ID=CRD42021246067, Identifier CRD42021246067.

## 1 Introduction

Alcohol-related liver disease (ALD) is a chronic disease caused by long-term excessive drinking, it usually presents as fatty liver in the early stage and then progresses to alcoholic hepatitis (AH), liver fibrosis, liver cirrhosis, liver cancer, and severe alcoholic hepatitis (SAH) in severe cases (Crabb et al., 2020). ALD has a high mortality rate, accounting for 0.9% of global deaths and 47.9% of deaths from liver cirrhosis (Sarin et al., 2019). There is no national epidemiological data on ALD in China, but the regional epidemiological survey shows that the prevalence and mortality of ALD are increasing year by year, which is the second major cause of liver injury after viral hepatitis (Chinese Medical Association et al., 2019). Active management and treatment of ALD are important measures that could influence the long-term consequences for patients. The current standard of care for ALD includes continuous abstinence (Veldt et al., 2002), supplement nutrition and vitamins (European Association for the Study of the Liver. Electronic address: easloffice@easloffice.eu and European Association for the Study of the Liver, 2018), liver protection and anti-inflammatory according to the stage of the disease, actively preventing complications, and preventing the occurrence and the progress of liver damage (Suk et al., 2014; Wen et al., 2021). For patients with SAH, a steroid is an option for treatment, but it is only up to one-third of patients are eligible for steroids (Shasthry, 2020). Besides, a portion of patients do not respond and its long-term benefit in those who respond to steroids in the short-term is doubtful (Thursz and Morgan, 2016; Mitchell et al., 2020). Liver transplantation is the final treatment option, but many SAH patients are ineligible for liver transplantation. Therefore, new therapeutic methods need to be explored (Sarin et al., 2019). With the insight into the gut-liver axis, researchers pay more and more attention to the role of the intestinal microbiome in ALD. Studies have found that in ALD, the number and the composition of the intestinal microbiota are disordered, and intestinal mucosal barrier function is damaged. Bacterial translocation and imbalance of intestinal microbiota will further aggravate liver injury (Li and Xing, 2019). These indicate that intestinal microbiota plays a critical role in the pathogenesis of ALD (Szabo and Bala, 2010; Seo and Shah, 2012). Microbiome-targeted therapies (MTTs) (Sharpton et al., 2019) are a method to manipulate the intestinal microbiota, which is expected to have a role in improving ALD (Chi et al., 2020). Recently, some narrative reviews of MTTS in the treatment of ALD have been published (Philips et al., 2017; 2017; 2018). However, these narrative reviews only qualitatively analyze and evaluate the relationship between intestinal microbiota and ALD, and have certain limitations and subjectivity, and some with small sample sizes and inconsistent results. It is necessary to conduct a meta-analysis to quantitatively evaluate the clinical consistency of multiple research results, and evaluate the effect indicators more accurately, strictly, and objectively. Overview of the pieces of literature, we found some articles are about MTTs on ALD treatments, which include 10 articles on probiotics (Loguercio et al., 2005; Lata et al., 2007; Kirpich et al., 2008; Stadlbauer et al., 2008; Koga et al., 2013; Kwak et al., 2014; Han et al., 2015; Macnaughtan et al., 2020; Zhang et al., 2020; Li et al., 2021), 6 articles on antibiotics (Madrid et al., 2001; Vlachogiannakos et al., 2009; Bass et al., 2010; Kalambokis et al., 2012; Vlachogiannakos et al., 2013; Madsen et al., 2018), 3 articles on fecal microbiota transplantation (FMT) (Philips et al., 2017; 2017; 2018), and only one on synbiotics (Liu et al., 2004). However, according to the strict inclusion and exclusion criteria, only 5 RCTs on probiotics were included in this review. The methods recommended by the international Cochrane Collaboration were used to carry out a meta-analysis of the effect of MTTs on the treatment of ALD and the relation between MTTs and body immunity. We hope this review could provide some evidence-based medical data for the treatment of ALD.

## 2 Methods

The search strategy, eligibility criteria, and outcomes were registered on the PROSPERO website (Registration link: https://www.crd.york.ac.uk/prospero/; Registration number: CRD42021246067).

### 2.1 Search strategy

A systematic search strategy was utilized to browse through electronic databases including the Cochrane Library, Medline/PubMed, Embase, CNKI, Wanfang Data, Weipu Database, and other databases, the clinical RCT studies on the MTTs in ALD were manually identified from January 2000 to May 2021. The search strategy adopted the combination of subject words and free words to cover the following terms: “Probiotic,” “Prebiotic,” “Synbiotic,” “Fecal Microbiota Transplantation,” “Bacteriophage,” “Antibiotic,” “Alcoholic Liver Disease,” “Alcohol-related liver disease,” “Alcoholic Hepatitis,” “Alcoholic cirrhosis,” and “Alcoholic Steatohepatitis”. Taking PubMed as an example, the search strategy is shown in Table 1. If necessary, trace back to the reference lists from potentially relevant papers and previous review articles to obtain more comprehensive studies and data.

**TABLE 1:** Search strategy.

#1 Probiotic OR Prebiotic OR Synbiotic OR Fecal Microbiota Transplantation OR Bacteriophage OR Antibiotic	#3 # 1 AND #2
#2 Alcoholic Liver Disease OR alcohol-related liver disease OR Alcoholic Hepatitis OR Alcoholic cirrhosis OR Alcoholic Steatohepatitis	

### 2.2 Inclusion and exclusion criteria

Inclusion criteria were as follows: Randomized controlled trials (RCT) with ALD patients of any gender, age, or race who were diagnosed by serum liver enzymes, imaging techniques (mostly ultrasonography), or liver biopsy. Exclusion criteria were as follows: Literature is not in Chinese or English; The literature types include review, conference report, case report; Repeated publications, obvious errors in the original data, and articles that are not available in full; Animal experiment.

### 2.3 Assessment of outcomes

Referring to the outcome indicators observed in previous studies, we chose alanine aminotransferase (ALT), aspartic transaminase (AST), γ-glutamyl transpeptidase (GGT), total bilirubin (TBIL), and prothrombin time (PT) as the primary outcome indicators, and tumor necrosis factor-α (TNF-α), interleukin-6 (IL-6), the number of *Escherichia coli* and *Bifidobacteria* in the intestinal microbiota as the second outcome indexes. Trials included in this review must have one of the above clinical indicators. In addition, the safety of the included studies will be assessed in our meta-analysis. Since the long-term prognostic indicators, MELD score, and Child-pugh score were not evaluated in RCTs, these outcome data were not analyzed in the current review.

### 2.4 Data extraction and quality evaluation

Using a pre-designed information extraction form (Supplementary material Appendix S1), the data was extracted and cross-checked. Two researchers (C.X. and S.X.) independently screened the literature, extracted data, and evaluated the quality by the mutual blind method. If the selections from two researchers were not consistent or had discrepancies, seek the third professor (X.H.C) to evaluate it and help make the final decision. After the relevant literature was retrieved by the authors, the Endnote document management software was used to sort out the literature. The authors reviewed the titles and abstracts of the literature, selected the relevant literature based on the inclusion and exclusion criteria, and preliminarily excluded articles that were inconsistent with the research purpose. Then, the authors read the full text carefully to determine the final inclusion of the publications. The risk of bias was assessed according to the Cochrane Risk of Bias Table (Higgins et al., 2011). The main assessment indicators included randomization, allocation concealment, blinding of participants and personnel, outcome data integrity, selective reporting, and other sources of bias. If the information is incomplete, we can contact the author to supplement the required information.

### 2.5 Statistical analysis

**FIGURE 1:**
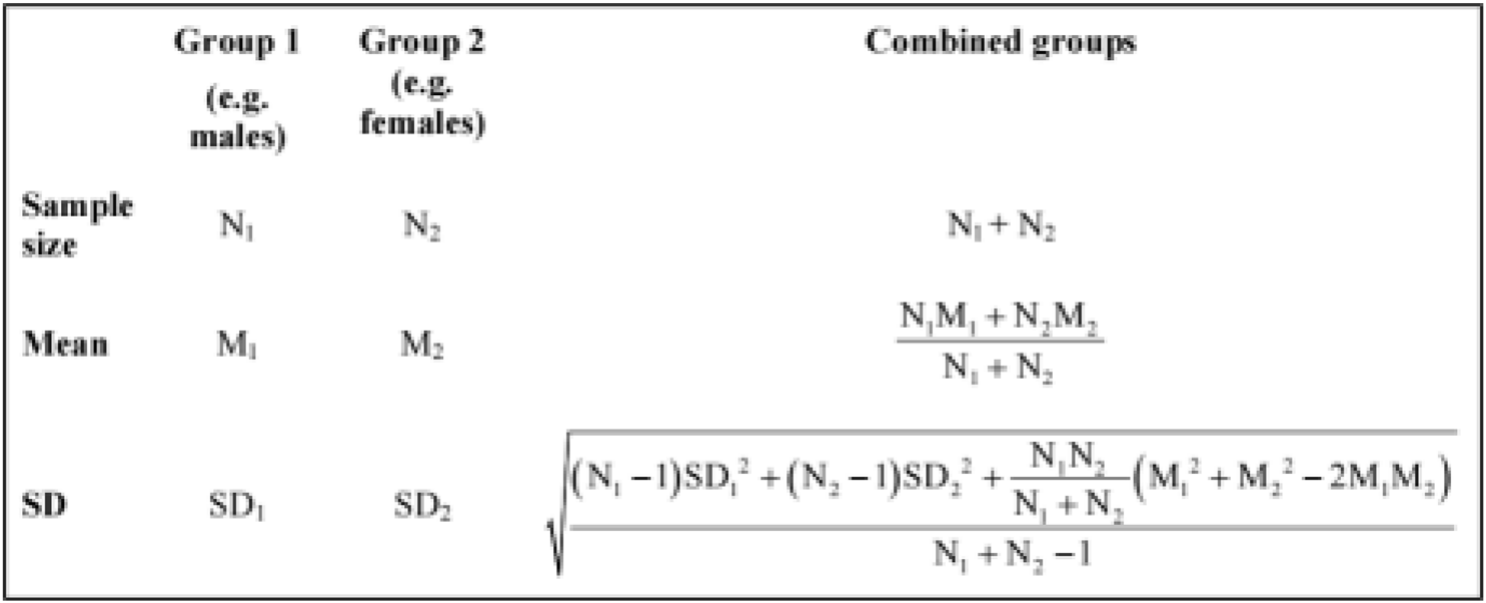
The calculation formula of the combined groups.

Statistical analysis of the included literature was performed using RevMan 5.4 software provided by the Cochrane Collaboration. The following items interested in this study were assessed: ALT, AST, GGT, TBIL, TNF-α, IL-6, intestinal *Escherichia coli*, and *Bifidobacteria.* The data extracted in these outcomes were continuous variables (numerical variables), mean difference (MD), and 95% confidence interval (95% CI) were combined into an effect size. However, when the measurement units are inconsistent, the standardized mean difference (SMD) and 95% CI combined statistics were selected, and *p* ≤ 0.05 is considered statistically significant. Heterogeneity was analyzed by the χ^2^ test, and the magnitude of heterogeneity was represented by I^2^. If *p* > 0.05, I^2^ ≤ 50%, it can be considered that multiple studies are homogeneous, and the fixed effects model was used for analysis; If *p* ≤ 0.05, I^2^ > 50%, indicating large heterogeneity among studies, sensitivity analysis was conducted to find the cause of heterogeneity, if no source of heterogeneity was found, the random-effects model was used to calculate the combined statistics. When there were multiple intervention groups and one control group, multiple intervention groups were combined into one intervention group, and the data of each intervention group were combined into one sample size, mean, and standard deviation. According to the Cochrane Handbook for Systematic Reviews of Interventions (The Cochrane Collaboration. Higgins., 2008), when there are more than two groups to combine, the simplest strategy is to apply the calculation formula for combining groups as presented in Figure 1 (i.e., combine group 1 and group 2 to create group ‘1 + 2’, then combine group ‘1 + 2’ and group 3 to create group ‘1 + 2+3’, and so on).

## 3 Results

### 3.1 Search results

**FIGURE 2:**
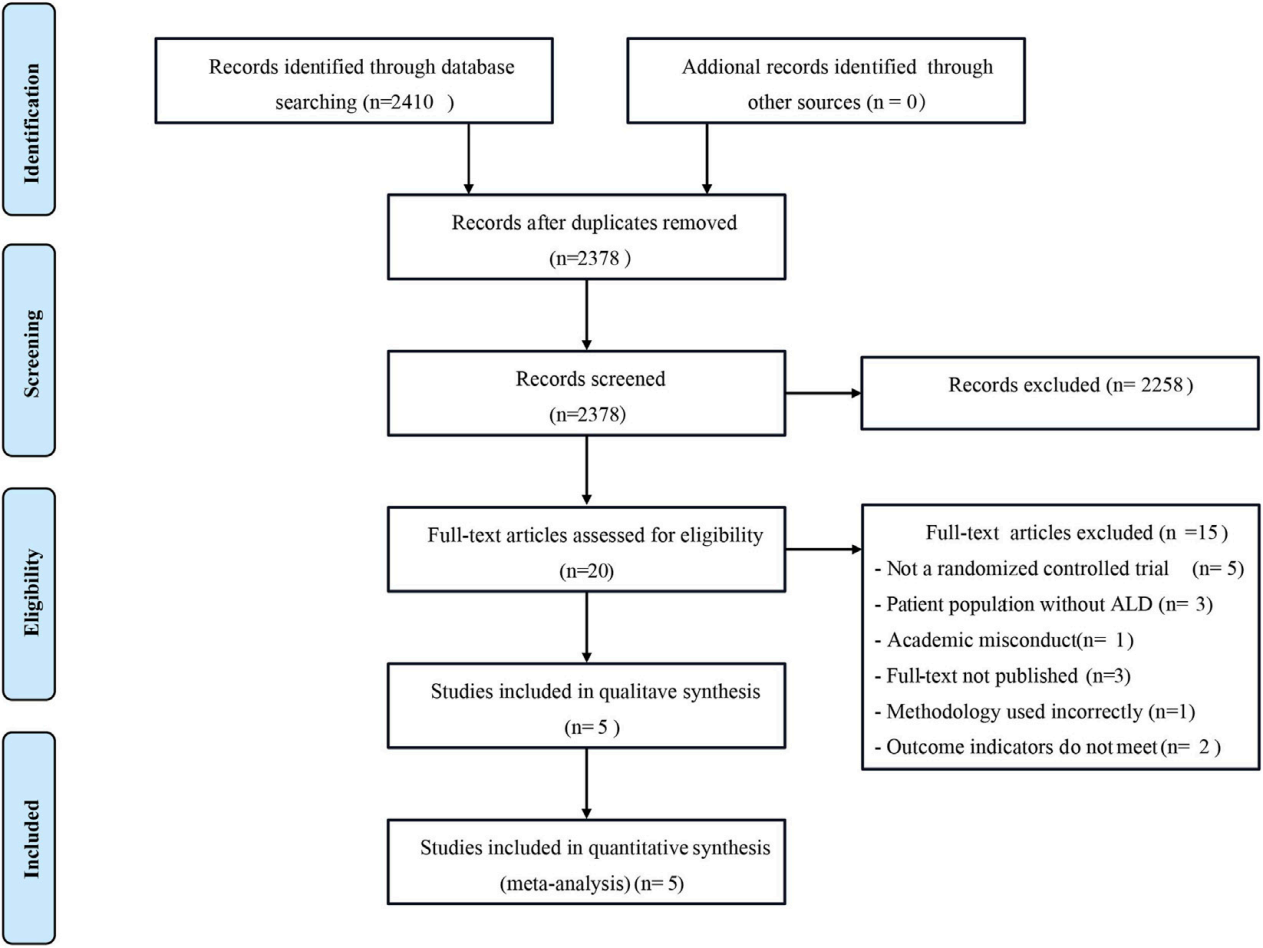
Flow diagram depicting the study selection process.

According to the search strategy provided by the Cochrane Collaboration, a total of 2,410 relevant literature were retrieved from the Chinese and English databases. After reading the titles and abstracts of the literature, 20 kinds of literature that could be included were initially screened out after excluding the articles that were completely inconsistent with the research content. After reading the full text carefully, 5 RCT studies (including 456 ALD patients) were finally included, 1 in Chinese and 4 in English. The literature screening process is shown in Figure 2, and the basic characteristics of the included pieces of literature are presented in Table 2.

**TABLE 2:** Basic characteristics of included studies.

Study	Country	Study object	Age	Sex	Total sample size		Intervention		Duration	Outcome	Risk of bias
					Experimental group	Control group	Experimental group	Control group			
Kirpich 2008 (Kirpich et al., 2008)	Russia	mild alcoholic hepatitis	Standard therapy:42.0 ± 2.18; Probiotic therapy:42.1 ± 1.89	male	32	34	Bififidobacterium bififidum and *Lactobacillus* plantarum 8PA3	Standard therapy	5d	ALT,AST,GGT,LDH,TB, *Escherichia coli*, Bifidobacterium,*Lactobacillus*, *Enterococcus*	High
Li 2021 (Li et al., 2021)	China	alcoholic fatty liver	30–65 years old	male	112	46	*Lactobacillus* casei		60d	ALT,AST,GGT,TBIL, Endotoxin,TG,TC,LDLC, HDLC,IL-1β,TNF-α,IL-6,IL-10, *Escherichia coli*, *Enterococcus*, *Bacteroides fragilis*, Bififidobacterium longum, *Lactobacillus* acidophilus, *Clostridium* leptum	Low
Koga 2013 (Koga et al., 2013)	Japan	alcoholic liver cirrhosis	Y400 group: 52.6 ± 11.8; Placebo group: 53.9 ± 14.9	(male:female) Y400 group: 15:3; Placebo group:15:4	18	19	probiotic beverage Yakult 400 (Y400) (*Lactobacillus* casei strain Shirota)	Placebo	4w	Bififidobacterium, Prevotella, *Clostridium*, Veillonella, *Fusobacterium*, Enterobacteriaceae, *Lactobacillus* casei, Shirota	Low
Han 2015 (Han et al., 2015)	South Korea	mild alcoholic hepatitis	All patients:52.7 ± 11.3	Male [n (%)]:75 (64)	60	57	*Lactobacillus* subtilis/*Streptococcus* faecium	Placebo + Standard therapy	7d	TP,Alb,AST,ALT,ALP,GGT,TB,TCHO,PT,TNF-α,IL-1β,LPS, *Escherichia coli*, *Enterococcus*	Low
Zhang 2020 (Zhang et al., 2020)	China	mild alcoholic injury	control group:46.72 ± 3.98; Experimental group: 47.43 ± 4.22	(male: female) control group:30:19; Experimental group:32:17	39	39	Tetralogy of viable bifidobacterium tablets	Standard therapy	2w	ALT, AST,GGT,TNF-α,IL-6,hs-CR,*Enterococcus*, *Lactobacillus*,Bifidobacterium, *Escherichia coli*	Unclear

### 3.2 Literature quality assessment

**FIGURE 3:**
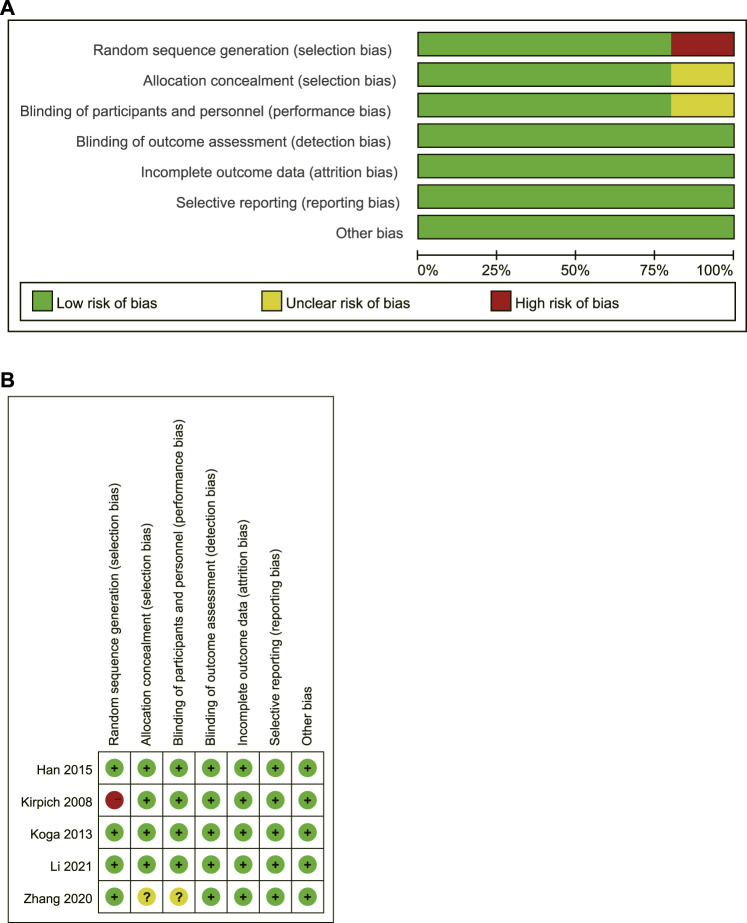
Risk assessment of bias in included studies **(A)** A review of the authors’ judgments about each risk of bias item presented as percentages; **(B)** A review of the authors’ judgments about each risk of bias item for included studies. Vertical represents multiple studies corresponding to a bias, while horizontal represents multiple bias evaluations corresponding to a study.

According to the Cochrane Risk of Bias table, the bias risk of the included pieces of literature was assessed. One of the pieces of literature (Kirpich et al., 2008) had a high bias risk due to the lax random method (according to the date of study entry). In the other 4 studies, three (Koga et al., 2013; Han et al., 2015; Li et al., 2021)showed a low risk of bias, and one (Zhang et al., 2020) showed an unclear risk of bias. The bias risk assessment is shown in Figure 3.

### 3.3 Outcome analysis

#### 3.3.1 Chemistry indicators related to liver injury

**FIGURE 4:**
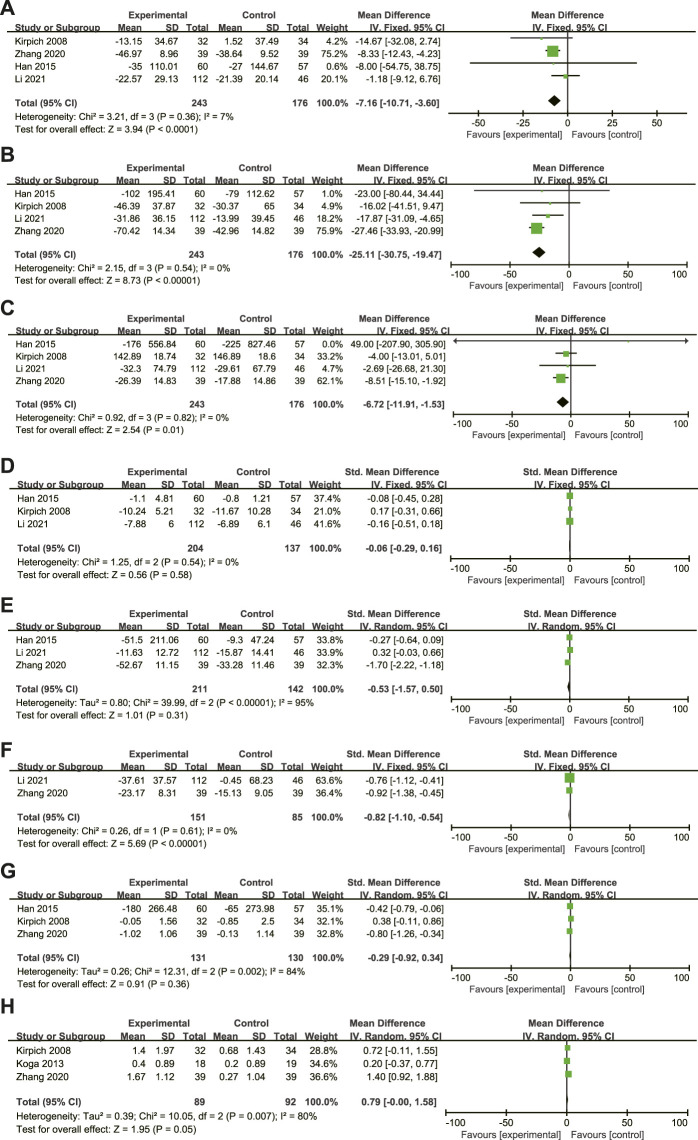
**(A–H)**: Effect of microbiome-targeted therapies on ALT, AST, GGT, TBil, TNF-α,IL-6, Escherichia coli and Bifidobacteria in ALD patients. Each block represents the weight of each study, and the larger the weight, the larger the area of the block; The length of the line segment represents the 95% confidence interval of each study effect; The diamond represents the summary results of meta-analysis and synthesis of various studies; The diamond width represents the 95% confidence interval of the aggregated effect quantity.

A total of 4 articles (Kirpich et al., 2008; Han et al., 2015; Zhang et al., 2020; Li et al., 2021) compared the changes in ALT, AST, and GGT levels of ALD patients before and after treatment, including 419 ALD patients (243 in the experimental group and 176 in the control group). There was homogeneity among the studies (ALT: I^2^ = 7%, *p* = 0.36; AST: I^2^ = 0%, *p* = 0.54; GGT: I^2^ = 0%, *p* = 0.82.), and the fixed-effects model was used for analysis (Figures 4A–C). The results showed that the difference between the two groups was statistically significant (ALT: MD = −7.16, 95% CI: 10.71∼-3.60, *p* < 0.0001; AST: MD = −25.11, 95% CI: 30.57∼-19.47; *p* < 0.00001; GGT: MD = −6.72, 95% CI: 11.91∼-1.53; *p* = 0.01), and the MTTs can significantly reduce the ALT, AST, GGT levels of ALD patients.

A total of 3 articles (Kirpich et al., 2008; Han et al., 2015; Li et al., 2021) compared the changes in TBIL levels in ALD patients before and after treatment, including 341 ALD patients (204 in the experimental group and 137 in the control group). To eliminate the difference in measurement units between studies, SMD is used. The studies are homogeneous (*p* = 0.54, I^2^ = 0%), and the fixed effects model is used for analysis (Figure 4D). The results showed that there was no statistically significant difference between the two groups (SMD = −0.06, 95% CI: 0.29–0.16; *p* = 0.58). This may be due to the low levels of TBIL in the study subjects at admission, as well as the short duration of probiotic treatment and weak efficacy, thus underestimating the role of probiotics.

Only one study (Han et al., 2015) evaluated the effect of probiotics intervention on PT prolongation in ALD patients, which showed that the prolongation of PT was improved after therapy with probiotics (*p* = 0.039). Since there is only one study, meta-synthesis cannot be carried out.

#### 3.3.2 Inflammatory indicators

A total of 3 articles (Han et al., 2015; Zhang et al., 2020; Li et al., 2021)compared the changes in TNF-α levels before and after treatment in ALD patients, including 353 ALD patients (211 in the experimental group and 142 in the control group). To eliminate the difference in measurement units between studies, SMD is used. There was great heterogeneity among studies (*p* < 0.001, I^2^ = 95%), but no source of heterogeneity was found after sensitivity analysis, indicating low sensitivity and relatively robust and reliable results. The existence of heterogeneity may be related to differences including population ethnicity, disease state, intervention measures, and treatment course in the three included articles. These are included in the study itself and cannot be subjectively excluded, so the random effects model can be used for analysis (Figure 4E). The results showed that there was no statistically significant difference between the two groups (SMD = −0.53, 95% CI: 1.57–0.50; *p* = 0.31).

A total of 2 articles (Zhang et al., 2020; Li et al., 2021) compared the changes in IL-6 levels in ALD patients before and after treatment, including 236 ALD patients (151 in the experimental group and 85 in the control group). To eliminate the difference in measurement units between studies, SMD is used. The studies are homogeneous (*p* = 0.61, I^2^ = 0%), and the fixed effects model is used for analysis (Figure 4F). The results showed that the level of IL-6 in the experimental group was significantly lower than that in the control group (SMD = −0.82, 95% CI: 1.10∼-0.54; *p* < 0.00001). (SMD = −0.82, 95% CI: 1.10∼-0.54; *p* < 0.00001).

#### 3.3.3 Intestinal microbiota

A total of 3 articles (Kirpich et al., 2008; Han et al., 2015; Zhang et al., 2020) compared the changes in intestinal *Escherichia coli* levels in ALD patients before and after treatment, including 261 ALD patients (131 in the experimental group and 130 in the control group). To eliminate differences in units of measurement between studies, SMD was used. There was heterogeneity among the studies (*p* = 0.002, I^2^ = 84%), this may be related to some differences in ethnicity, dietary habits, disease status, length of treatment and intervention dose, and type of probiotics in the studies we included. In addition, the blind method of trial design and follow-up time were different, and the random-effects model was used for analysis (Figure 4G). The results showed that there was no significant difference between the two groups (SMD = −0.29, 95%CI: 0.92-0.34; *p* = 0.36).

A total of 3 articles (Kirpich et al., 2008; Koga et al., 2013; Zhang et al., 2020) compared the changes in the levels of *Bifidobacteria* in ALD patients before and after treatment, including 181 ALD patients (89 cases in the experimental group and 92 cases in the control group). There is heterogeneity among the studies (*p* = 0.007, I^2^ = 80%), the presence of heterogeneity may differ from race, dietary habits, disease status, length of treatment, intervention dose, and the blind method of trial design in the three included studies, and the random-effects model was used for analysis (Figure 4H). The results showed that the number of *Bifidobacteria* in the experimental group was significantly higher than that in the control group (MD = 0.79, 95% CI: 0.00–1.58; *p* = 0.05), but it was on the edge of statistical significance, and further analysis after expanding the sample size was needed. (MD = 0.79, 95% CI: 0.00–1.58; *p* = 0.05).

### 3.4 Safety assessment

Only one article (Zhang et al., 2020)) evaluated the adverse reactions in the study. The experimental group and control group experienced adverse reactions such as stomach discomfort, constipation, and nausea, and the incidence of adverse reactions in the experimental group and the control group was 8.16% and 4.08% respectively, with no statistically significant difference (*p* = 0.399). This indicates that microbiome-targeted therapies have fewer adverse reactions and a higher safety factor.

### 3.5 Sensitivity analysis

TNF-α, intestinal *E. coli*, and Bifidobacterium outcome indicators were tested for heterogeneity before statistical analysis. The results showed that there was significant heterogeneity among the studies, so the random-effects model was used. When the statistics of each indicator were combined, the single study included was eliminated one by one, and then the indicators were combined again for analysis. The statistical results of each time were compared with the statistical results before the elimination, and the results showed that there was no significant difference, indicating that the sensitivity was low, and the results were stable and reliable.

### 3.6 Publication bias

The number of articles included in each index of this study is less than 10, and publication bias has not been assessed. However, in the process of literature retrieval, multiple databases were searched in strict accordance with the criteria for inclusion and sorting to ensure accurate retrieval, which can reduce publication bias caused by missing literature.

## 4 Discussion

The management and treatment of ALD have become an important public health issue, and at present, new therapeutic targets have begun to target the pathophysiological mechanism of intestinal microbiome disorders (Crabb et al., 2020). There have been some studies targeting intestinal microbiome therapy, but the conclusions are not all consistent, and no relevant meta-analysis has been reported. In this systematic review and meta-analysis, a total of 5 RCT studies were included to explore the therapeutic effect of MTTs on ALD. Meta-analysis showed that the MTTs significantly reduced the levels of serum ALT, AST, GGT, and IL-6 in patients with ALD, and increased the number of intestinal *Bifidobacteria* but did not improve the level of TBIL, TNF-α, and *E. coli*.

Serum ALT, AST, GGT, TBIL, and PT are common indexes of liver injury. Some experimental studies (Segawa et al., 2008; Fang et al., 2019; Nam et al., 2019; Gu et al., 2020; Seo et al., 2020; Zheng et al., 2020; Hsieh et al., 2021) show that probiotics supplementation can reduce the levels of liver enzymes such as ALT and AST by enhancing the integrity of the intestinal barrier and restoring intestinal homeostasis in ALD mice, which indicated the improvement of alcohol-induced liver injury. Regulating the intestinal microbiota can improve intestinal permeability, maintain the integrity of the gut barrier, reduce liver inflammation, and alleviate liver damage (Chi et al., 2020). Patients with alcohol-related liver disease can use microbial preparations and fecal microbiota transplantation to regulate dysregulated gut microbiota to achieve therapeutic effects such as reducing liver enzyme indicators and inflammatory factors. The results of our meta-analysis showed that ALT, AST, and GGT levels could be reduced, which was also consistent with the RCT of the largest sample size and longest duration (Li et al., 2021), but the reduction of TBIL was not significantly different between the treatment group and the control group. This may be due to the level of TBIL in the included subjects (Kirpich et al., 2008; Han et al., 2015) being only mild elevation, and the probiotic intervention time was short. PT can reflect liver synthesis and reserve function and is a sensitive index to judge the severity of liver disease. Han’s study (Han et al., 2015) indicated that probiotics were effective in improving PT prolongation in patients with AH (excluding severe AH), suggesting that probiotic intervention may be used to improve the prognosis of ALD. At present, MTTs used in clinical studies or basic experiments include probiotics, prebiotics, antibiotics, FMT, and/or phages. However, the only probiotic intervention was found in RCTs. Therefore, the intervention in these 5 articles is all probiotic.

IL-6 and TNF-α, produced by activated Kupffer cells are important inflammatory cytokines, which could induce liver inflammation and promote the occurrence and progression of ALD (Kitazawa et al., 2003; Shi et al., 2017). Intestinal microbiota disturbances and lipopolysaccharide (LPS) overload could mediate the activation of Kupffer cells in the liver (Chi et al., 2020). Our results showed that the level of IL-6 decreased significantly after MTTs treatment, but the difference in TNF-α level was not statistically significant, which was inconsistent with many other ALD mice studies (Segawa et al., 2008; Wang et al., 2013; Kim et al., 2018; Seo et al., 2020; Zheng et al., 2020; Hsieh et al., 2021; Li et al., 2021). Studies on ALD mice suggest that probiotics can reduce the level of TNF-α, alleviate alcohol-induced oxidative stress, and improve the degree of liver inflammation (Segawa et al., 2008; Wang et al., 2013; Kim et al., 2018; Seo et al., 2020; Zheng et al., 2020; Hsieh et al., 2021; Li et al., 2021). However, in human studies, the results are controversial. Han (Han et al., 2015) showed a statistically significant decrease in TNF-α levels after probiotics treatment (compared with placebo, *p* = 0.042). Other studies showed that the level of TNF-α decreased significantly with the high-dose probiotic treatment, but there was no significant difference in the low-dose probiotic group (Li et al., 2021). The dosage of probiotics may be one of the factors affecting its effect on the TNF-α level. In addition, in Tandon, et al.'s study, probiotic VSL # 3 showed a slight increase in serum TNF-a levels in patients with compensatory liver cirrhosis, which may be related to the limitations of the small sample size and nonrandomized control of the study (Puneeta et al., 2009). Therefore, in the future, a larger scale and longer duration RCTs will be needed to verify the effect of MTTs in improving inflammation in ALD patients.

*Bifidobacteria* belong to *Actinobacteria*, a Gram-positive bacterium that plays a beneficial role in intestinal homeostasis in the human body. It can produce bacteriocins and organic acids, regulate intestinal mucosal immunity, and resist the invasion and colonization of intestinal pathogens. It is often used as a probiotic agent and has potential benefits in many intestinal diseases as well as extra-intestinal diseases (Tojo et al., 2014; Binda et al., 2018; Wang et al., 2018.). Our results showed that probiotics could increase the number of *Bifidobacteria*, which was consistent with the results of Kirpich (Kirpich et al., 2008), Li (Li et al., 2021), and Zhang (Zhang et al., 2020), but Koga’s study (Koga et al., 2013) showed that the number of *Bifidobacteria* did not change after probiotics therapy, which may be due to the type of probiotics in the intervention measures. Most probiotics are *Lactobacillus*, *Lactobacillus rhamnosus*, *Bifidobacterium*, or other combination preparations (Li et al., 2016), The probiotics involved in this study were *Bifidobacterium*, *Lactobacillus casei*, and *Bacillus subtilis*/*Streptococcus faecium,* with different doses for each study (Supplementary Material Appendix S2). A different dosage, different species, and different formulas of probiotics may have different effects on the human body. Some probiotics are effective at low levels of colony-forming unit (CFU), while others require higher levels of CFU, depending on the species (Sanders et al., 2010), However, due to the limitation of the number of included studies, subgroup analysis on the medication duration and the dose was not conducted in this meta-analysis. *Escherichia coli* is one of the most common pathogenic bacteria, and it is associated with liver inflammation, infection, and disease severity in patients with chronic alcohol consumption. (Chen et al., 2015.). Therefore, we can regulate intestinal microbiota by supplementing beneficial bacteria and reducing pathogenic bacteria to achieve the purpose of treating ALD.

Although the design and analysis of this research are strict, there are certain limitations of the original research. 1) First of all, some studies on the evidence-based method are not clear about the randomization method, the specific situation of the allocation concealment and the implementation of the blind method is not clear, and the overall quality of the included literature is not high, which will lead to implementation bias and measurement bias. Therefore, it is recommended that future RCTs should be performed with a detail random grouping, allocation concealment, and blind implementation methods as much as possible, which not only can be intuitive and credible but also makes the research more rigorous and convincing. 2) There are differences in the types, doses, and treatment duration of intervention methods in each study. 3) This meta-analysis only analyzes the changes in serum liver enzymes and other indicators, and cannot replace the direct assessment of liver statuses such as imaging techniques and liver biopsy. 4) We also acknowledged the potential variability from multiple data collection and measurement platforms, which could impact our results from data analyses. 5) Due to the small number of documents included in each analysis (less than 10), publication bias was not evaluated. However, this study is the first systematic review and meta-analysis of the effect of MTTs on ALD. It has strict retrieval and selection criteria, and quantitative synthesis of all existing relevant literature, and relatively reliable conclusions are obtained. It has a certain reference value and points out the direction for future research.

## 5 Summary

In conclusion, targeting the intestinal microbiome therapy can improve liver function indicators, and inflammatory factor levels and regulate intestinal microbiota in patients with ALD, which may be beneficial to ALD. However, the current number of clinical studies is small, and the duration of follow-up is short. Larger and more rigorously designed multi-center RCT studies are needed to further improve the research in this field and provide a reliable basis for clinical treatment.

## Data Availability

The original contributions presented in the study are included in the article/Supplementary Material, further inquiries can be directed to the corresponding authors.

## Abbreviations

ALD, Alcohol-related Liver Disease; MTTs, microbiome-targeted therapies; AH, alcoholic hepatitis; SAH, severe alcoholic hepatitis; FMT, fecal microbiota transplantation; RCT, randomized controlled trials; ALT, alanine aminotransferase; AST, aspartic transaminase; GGT, γ-glutamyl transpeptidase; TBIL, total bilirubin; PT, prothrombin time; TNF-α, tumor necrosis factor-α; IL-6,interleukin-6; MD, mean difference; SMD, standardized mean difference.
