# Supplementary material for: Intestinal microbiome-targeted therapies improve liver function in alcohol-related liver disease by restoring bifidobacteria: a systematic review and meta-analysis
Source: Front Pharmacol. 2024 Jan 8;14:1274261. doi: 10.3389/fphar.2023.1274261 (PMC10800551; doi:10.3389/fphar.2023.1274261)
Supplement: Supplementary file 1 [file Table1.DOCX]

**Supplementary material to the article, entitled “Effect of** **Intestinal Microbiome-targeted Therapies in Treatment of Alcohol-related Liver Disease: a systematic review and meta-analysis”**

**Appendix 1. Data extraction**

**Table S1. Information extraction form**

| Study | Country | Study object | Age | Sex | Total sample size | | Intervention | | Duration | Outcome | Risk of bias |
| --- | --- | --- | --- | --- | --- | --- | --- | --- | --- | --- | --- |
|  |  |  |  |  | experimental  group | Control group | experimental  group | Control group |  |  |  |
|  |  |  |  |  |  |  |  |  |  |  |  |

**Appendix 2.**

The concentration we know is the following： *Lactobacillus casei* at 80 billion Colony forming unit (CFU) /day for 4 weeks; 90 million CFU/day of *Bifidobacterium bifidum* and 900 million CFU/day of *Lactobacillus plantarum 8PA3* for 5 days; 10 billion or 20 billion CFU/day of *Lactobacillus casei strain Shirota* for 60 days; 50 million CFU/day of *Bifidobacterium* for 2 weeks.
